# Safety and Efficacy of Stereotactic Magnetic Resonance-Guided Adaptive Radiation Therapy (SMART) for Ultracentral Metastases in Non-Small Cell Lung Cancer

**DOI:** 10.1016/j.adro.2025.101906

**Published:** 2025-09-18

**Authors:** Elena Moreno-Olmedo, Ben George, Kasia Owczarczyk, David Woolf, John Conibear, Andy Gaya, Joss Adams, Luis Aznar-García, Timothy Sevitt, Peter Dickinson, Kevin Franks, Alex Martin, Veni Ezhil, Philip Camilleri, James Good, Crispin Hiley

**Affiliations:** a GenesisCare UK, Oxford & London, United Kingdom; b Oxford University Hospitals NHS Foundation Trust, Oxford, United Kingdom; c University College London, London, United Kingdom; d Cancer Research UK Lung Cancer Centre of Excellence, UCL Cancer Institute, London, United Kingdom

## Abstract

**Purpose:**

Stereotactic ablative radiation therapy (SABR) is a standard of care for early-stage lung cancer and thoracic oligometastatic or oligoprogressive disease. However, ultracentral lesions remain challenging because of their proximity to critical mediastinal structures and the associated risk of severe toxicity. Stereotactic magnetic resonance-guided adaptive radiation therapy (SMART) allows for daily plan adaptation and real-time tracking in breath-hold, enhancing target coverage while improving sparing of adjacent organs compared to conventional SABR.

**Methods and Materials:**

This retrospective study analyzed outcomes of SMART-based SABR for ultracentral metastatic lesions in patients with histologically confirmed non-small cell lung cancer (NSCLC). Ultracentral lesions were defined by planning target volume overlapping with the proximal bronchial tree, esophagus, or pulmonary vessels. Endpoints included grade ≥ 3 SMART-related toxicity, freedom from local progression, progression-free survival, and overall survival.

**Results:**

Between 2020 and 2023, 11 patients with 18 ultracentral NSCLC lesions underwent SMART. All treatments were delivered in breath-hold. The median dose was 40 Gy (range, 30-60 Gy) in 5 to 8 fractions. Online plan adaptation was performed for 100% of the 78 delivered fractions. No grade ≥ 3 toxicities were observed. Rates of grade 1 to 2 acute and late toxicities were 54% and 18%, respectively. At a median follow-up of 28 months (range, 5-41 months), 66.7% of patients were alive. One-year freedom from local progression was 93%. Median progression-free survival was 5.8 months (range, 1-39 months), and median overall survival was 20 months (range, 5-41 months).

**Conclusions:**

SMART with daily online adaptation achieved excellent local control and a favorable safety profile in ultracentral NSCLC, comparable to conventional non-adaptive SABR, but without severe toxicity.

## Introduction

For patients with non-small-cell lung cancer (NSCLC), stereotactic ablative body radiation therapy (SABR) is a standard of care for patients with early-stage cancer and for oligometastatic (OMD) or oligoprogressive disease (OPD).^1^, ^2^, ^3^

Although conventional SABR achieves excellent local control (LC), ultracentral tumor location makes the delivery of a safe effective treatment challenging. Contemporary series of nonadaptive conventional SABR for ultracentral tumors report LC rates of 83% to 90% at 2 to 3 years but at the expense of 7% to 34% of ≥G3 toxicity.^4^, ^5^, ^6^, ^7^ Stereotactic magnetic resonance-guided adaptive radiation therapy (SMART) enhances treatment precision. Daily online volume adaptation and plan reoptimization accounts for changes in tumor size and position relative to organs at risk (OARs). Real-time imaging permits treatment in breath-hold with automated gated beam delivery which eliminates the need for an internal target volume (ITV).

Current evidence for the benefit of SMART for ultracentral tumors is encouraging but is determined from cohorts of patients with heterogeneous primary cancer diagnoses.^8^, ^9^, ^10^, ^11^, ^12^, ^13^, ^14^, ^15^ Differences in tumor biology and systemic therapy regimes, in a heterogeneous cohort, may confound assessment of safety. We present our institution's experience of daily-SMART for ultracentral tumors, from a homogeneous cohort of patients with a primary diagnosis of NSCLC.

## Methods and Materials

### Study design and population

This single institution retrospective analysis assessed the safety and efficacy of SMART in ultracentral metastatic lesions from patients with histologically proven NSCLC. All patients provided written consent and were assessed by a tumor board. Ultracentral tumors were defined as any parenchymal lesion or lymph node whose PTV overlapped the proximal bronchial tree (PBT), esophagus or pulmonary vessels. OMD included ≤5 disease sites and OPD ≤ 3 progressing disease sites. Systemic therapy (ST) was held before SABR to avoid interactions with concurrent treatment.

The primary objective of this study was to assess the incidence of SABR related acute (<3 months) and late (>3 months) toxicity, graded according to the Common Terminology Criteria for Adverse Events version 5.0. A secondary objective was to evaluate time-to-event outcomes, measured from the first day of SMART. Freedom from locoregional progression (FFLP) was defined as the absence of progressive lung cancer within or adjacent to the radiation therapy field. Patients were censored at the time of death from distant metastases or at the last follow-up if alive without evidence of locoregional failure. Progression-free survival (PFS) was defined as the time from SMART initiation to the first occurrence of disease progression (locoregional or distant) or death from any cause. Patients lost to follow-up were censored at the date of last contact. Overall survival (OS) was defined as the time from SMART initiation to death from any cause, with patients lost to follow-up censored at the date of last known survival.

### SMART planning and delivery

Simulation consisted of a 0.35T magnetic resonance imaging (MRI) true fast imaging with steady-state free precession sequence and a computed tomography (CT) image. Simulations were performed in breath-hold at the same respiratory phase. The CT was deformably registered to the magnetic resonance (MR) to provide electron density information for dose calculation. A suitable tracking structure was identified through cine MR imaging to permit gated treatment. Contouring of gross target volume (GTV) and OARs was in concordance with national and international guidelines with mandatory peer review.^16^ The PTV margin was a 3-mm axial expansion and a 3- to 6-mm superior/inferior margin from the GTV.

A 6-MV photon fixed field SABR plan was generated with inverse planning with the following dosimetric aims: PTV V100% ≥ 95%, PTV V95 ≥ 98%, Dmax(0.1cc) ≥ 110% and ≤ 140%, with hotspots inside the GTV. PTV coverage was compromised, when necessary to meet OAR constraints with a minimum PTV V100% ≥ 70% required. SABR was delivered on a 6 MV 0.35T MRIdian (*ViewRay Systems, MRIdian*). A daily onset breath-hold MRI true fast imaging with steady-state free precession sequence scan was acquired for consideration of online adaptation. The contours (GTV and OARs within a 3-cm distance from PTV) were adapted daily. Plan optimization was performed to meet planning objectives and mandatory OAR constraints for each individual fraction.^16^ SABR was delivered with gated delivery during repeated breath-holds under continuous MR-guidance.

To estimate the reduction in PTV volume and overlap with OARs from SMART in breath-hold compared to conventional SABR with 4-dimensional CT, motion was simulated on the adaptive MR-Linac planning scan for each tumor. The mean range of motion in the superior-inferior, anterior-posterior, and left-right axis was applied as reported by the management of respiratory motion in radiation oncology report of American Association of Physicists in Medicine Task Group.^17^ A standard GTV-PTV margin expansion of 5 mm was then applied.

Institutional follow-up assessment was conducted 7 to 10 days after treatment. Further follow-up at our institution was not mandated but safety and efficacy outcomes were obtained from the referring oncologist.

### Statistical analysis

The Kaplan–Meier method was used to estimate FFLP, PFS, and OS, with a log-rank test for subgroup comparisons. The reverse Kaplan–Meier method was used to estimate the median follow-up duration, accounting for censoring. Paired *t* tests were used to assess the difference in PTV overlap with OARs between the conventional and adaptive approaches. Kruskal–Wallis test with post hoc pairwise comparisons using the Dunn's test was used for any other analyses.

## Results

Between September 2020 and August 2023, 11 patients with a histologically confirmed NSCLC received daily-SMART to treat a total of 18 thoracic lesions (Table 1). All patients had metastatic disease. Prior to SMART 4 patients had received a tyrosine kinase inhibitor for an actionable genomic alteration, 1 patient had received a tyrosine kinase inhibitor and chemotherapy, 2 patients had received immunotherapy, 1 patient had received chemotherapy, and 3 patients had not received any prior therapy in the metastatic setting (Table S1). During the peri-SMART period, the median time off-ST was 0.6 months prior SMART and 0.75 months following the last fraction.

**Table 1:** Demographics and clinic characteristics

Patients (lesions)	11 (18)
Age, median (range) y	67 (46-82)
**Gender, n (%)**	
Male	2 (18)
Female	9 (82)
**Lesion Type, n (%)**	
Lung parenchyma	2 (11)
Lymph node	16 (89)
**History of metastatic disease before SMART, n (%)**	
No	3 (27)
Yes	8 (73)
Thoracic metastasis	2 (18)
Extra-thoracic metastasis	6 (55)
**Histology subgroup, n (%)**	
Adenocarcinoma	8 (73)
Large cell neuroendocrine	3 (27)
**Ultracentral overlap, n (%)per lesions**	
PBT	7 (39)
PBT + pulmonary vessels	6 (33)
PBT + esophagus	1 (6)
Pulmonary vessels	2 (11)
PBT + pulmonary vessels+ esophagus	2 (11)

*Abbreviation:* PBT = proximal bronchial tree; SMART = stereotactic magnetic resonance-guided adaptive radiation therapy.

A summary of the dose prescriptions, targets volumes and dose coverage is presented in Table 2. The median SMART PTV volume overlapping the ultracentral OARs was 0.85 cm^3^ (8.4% of the PTV; range, 0.09-3.30 cm^3^). To meet OAR constraints 74% of tumors had prescriptions with BED_10_ *<* 100 Gy with a median PTV V100(%) of 92.5%.

**Table 2:** Treatment and dosimetric parameters

**Treatment prescriptions, median (range)**			
Dose prescribed, Gy		40 (30 - 60)	
Dose prescribed BED_10_, Gy		60 (48 – 105)	
Fractions prescribed		8 (5-8)	
**Fractions delivered and adapted**		73 (100%)	
**SMART fractionation schedules, n per lesion (%)**			
30 Gy/5#		4 (22.2)	
40 Gy/5#		4 (11.1)	
40 Gy/8#		5 (38.9)	
50 Gy/8#		1 (5.6)	
60 Gy/8#		4 (22.2)	
**Baseline SMART plan dosimetry, median (IQR)**			
GTV volume, cc		2.7 (11.2)	
PTV volume, cc		10.1 (23.7)	
PTV V100(%), cc		9.7 (23.8)	
PTV V100(%), %		92.5 (17.7)	
	**Gy**	**BED10 Gy**	
PTV D(99%)	34.3 (9.2)	49.1 (11.5)	
PTV D(98%)	35.4 (9.7)	51.7 (11.8)	
PTV D(95%)	38.5 (10.0)	57.2 (12.8)	
PTV Dmax, %		141.7 (2.4)	
PTV D(50%), Gy		47.7 (13.4)	
Prescription Dose Spill		1.2 (0.1)	
Modified gradient index		6.6 (3.9)	
**Target volume and ultracentral organ at risk overlap, median (IQR)**			
SMART PTV volume, cc		10.1 (23.7)	
Conventional-SABR PTV volume, cc		30.4 (23.7)	
SMART PTV overlap, cc		0.85 (0.9)	
Conventional-SABR PTV overlap, cc		4.7 (3.2)	
**Organ at risk doses, median (IRQ)**			
	**Gy**	**EQD2 α/β = 3**	
Esophagus D(0.1 cc)	27.1 (15.3)	34.6 (33.6)	
Esophagus Dmax	30.6 (16.6)	41.8 (38.9)	
Proximal bronchial tree D(0.1 cc)	33.5 (11.4)	62.7 (37.3)	
Proximal bronchial tree Dmax	37.1 (12.3)	68.4 (38.9)	
Lungs minus GTV V(20 Gy), %		1.8 (7.3)	
Lungs minus GTV V(5 Gy), %		22.1 (17.4)	
Lungs minus GTV D(mean), Gy		3.2 (3.7)	

*Abbreviations:* α/β = alpha/beta ratio; BED = biological effective dose; CTV = clinical target volume; EQD2 = equivalent dose in 2 Gy fraction; GTV = gross target volume; IQR = interquartile range; LR = left-right; PA = posterior-anterior; PTV = planning target volume; SABR = stereotactic ablative radiation therapy; SI = superior-inferior; SMART = stereotactic magnetic resonance-guided adaptive radiation therapy.

Conventional-SABR PTV = created as [gross target volume + (0.8 cm SI + 0.4 cm PA + 0.4 cm LR) = CTV] + 0.5 cm isotropic expansion.

**Figure 1:**
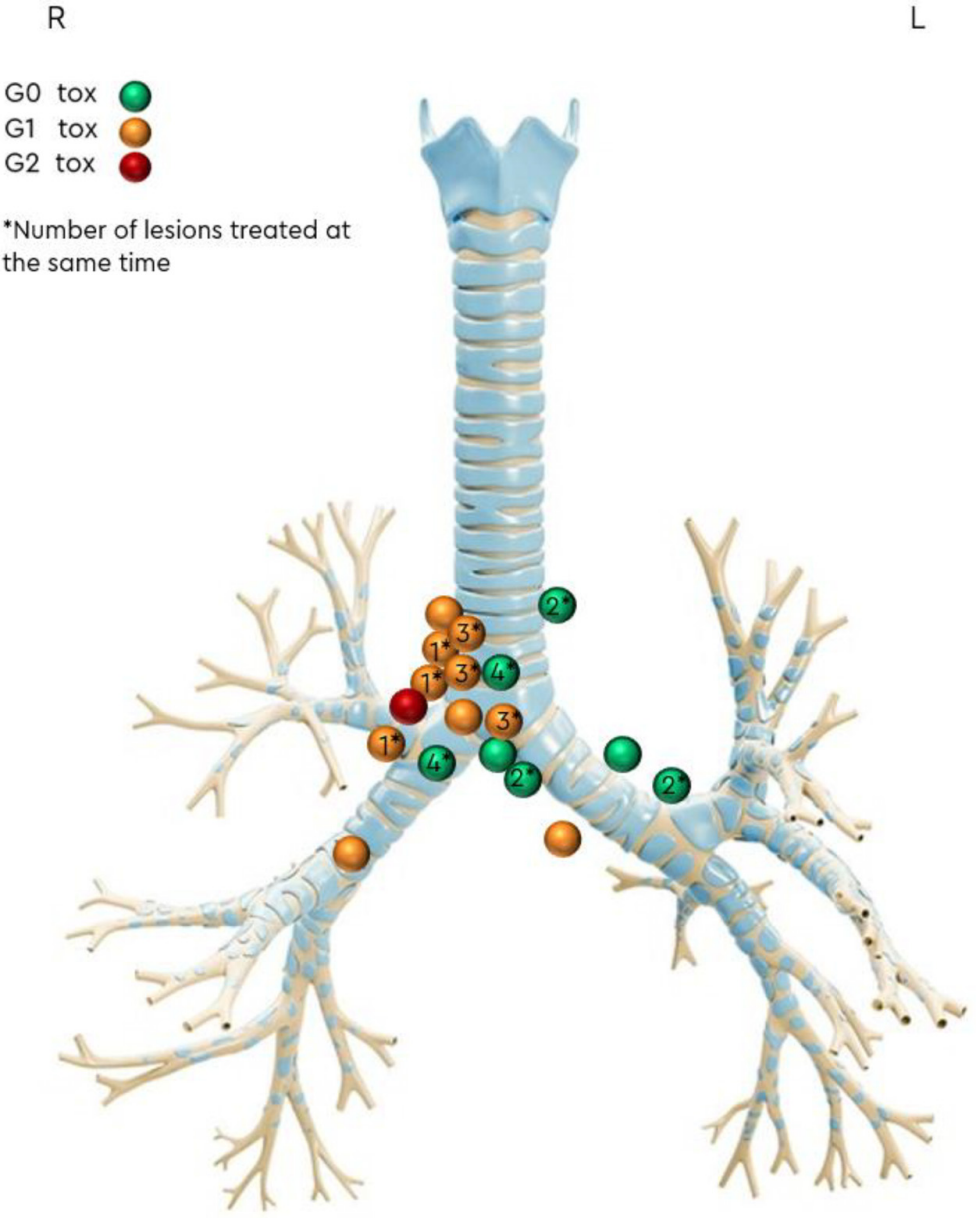
Distribution of each lesion location in relation to the PBT, graded by maximum reported toxicity and indicating those treated synchronously. *Abbreviation:* PBT = proximal bronchial tree.

The cumulative incidence of acute and late grade 1 to 2 toxicity was 54% and 18%, respectively. No grade 3 to 5 acute or late toxicities were reported (Table 3). Figure 1 represents the location of each lesion in relation to the PBT, graded by maximum reported toxicity and indicating those treated synchronously.

**Table 3:** Toxicity according to CTCAE v5

**Acute, n (%)**	6 (54.5)
G1	5 (45)
Fatigue	4 (36)
Cough	1 (9)
Dysphagia	1 (9)
Breathlessness on exertion	1 (9)
G2	1 (9)
Nausea/Vomiting	1 (9)
G3-5	0 (0)
**Late, n (%)**	2 (18)
G1	
Breathlessness on exertion	2 (18)
G3-5	0 (0)

*Abbreviation:* CTCAE = Common Terminology Criteria for Adverse Events.

**Figure 2:**
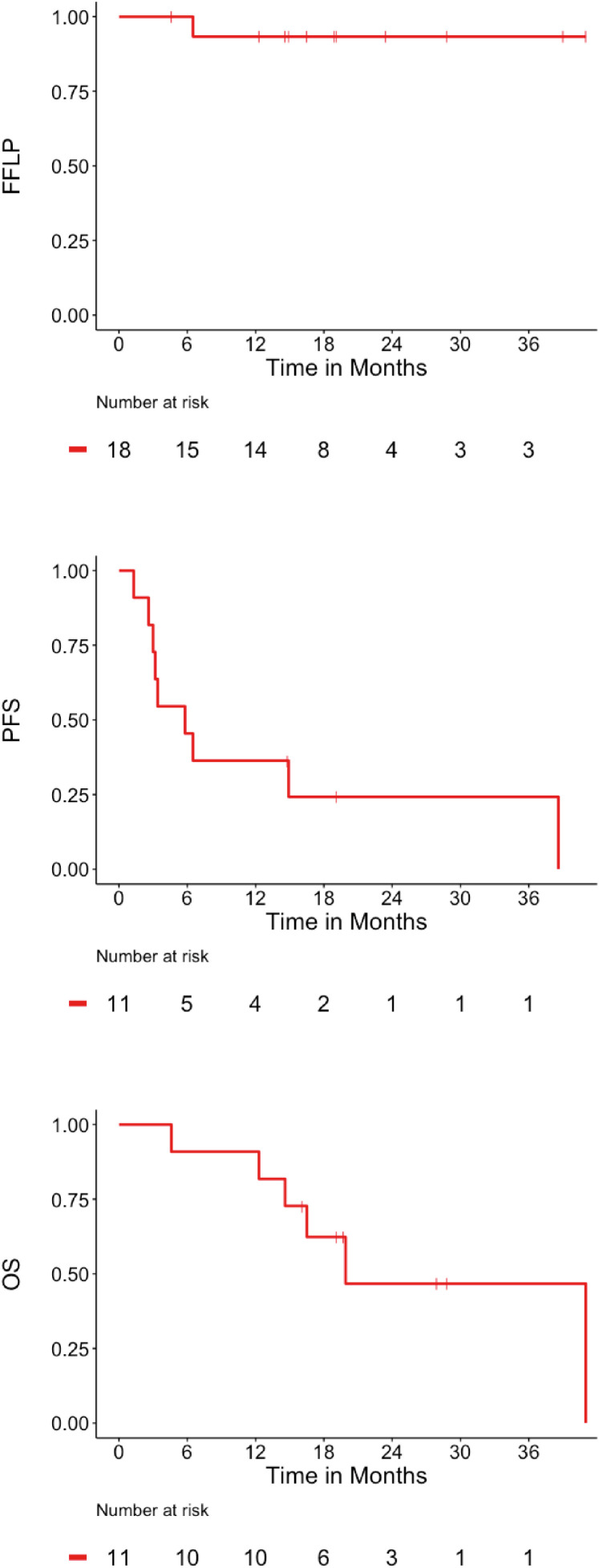
Kaplan–Meier function for freedom from local progression (FFLP), progression-free survival (PFS), and overall survival (OS). *P* values indicate a log-rank analysis between groups.

With a median follow-up of 28 months (range, 5 - 41 months), 1 lesion recurred within the SMART field. In this case the volume of overlap of the PTV with the left PBT and pulmonary vessels was 7.6 cm^3^. Consequently, the PTV coverage was compromised to meet OAR constraints, resulting in a PTV V100(%) of 71%. The 12-month FFLP rate was 93%. Intrathoracic but out of field recurrence was the primary pattern of failure in this cohort (42%), whereas 25% of the cohort had a distant relapse. Median PFS was 6 months (range, 1-39 months). Median and 1-year OS was 20 months (range, 5-41 months) and 91%, respectively (Fig. 2). These data are presented in the context of recently published studies on ultracentral lesions (Table 4).^4^, ^5^, ^6^, ^7^^,^^10^^,^^12^, ^13^, ^14^, ^15^

**Table 4:** Key outcomes compared to the most relevant reports of ultracentral tumours

Platform/ RMM	Adaptive approach, MRI-Linac (MRIdian®) Tracking, Gating						Non- Adaptive approach, CT-based 4D-CT/ITV			
	Current cohort	SMART Phase I (10)	Sandoval (12)	Regnery (14)	Dana-Farber (13)	La Rosa (15)	HILUS (6)	SUNSET (4)	Sunnybrook (5)	LUSTRE Phase 3** (7)
**UC definition**	PTV overlaps/ touch PBT and/or oesophagus and/or pulm vessels	GTV abutting mainstem bronchi, carina, or mediastinum	Per the HILUS trial definition	PTV overlaps PBT or oesophagus	GTV abutting PBT, oesophagus and/or great vessels	PTV overlaps Trachea, mainstem bronchi or oesophagus	1cm from PBT (GroupA) ≤1cm from lobar bronchi (GroupB)	PTV overlaps/ touch PBT and/or oesophagus and/or pulm vessels	PTV overlap/ touch PBT and/or oesophagus	Central 1 cm of mediastinum or 2 cm of PBT (No provide UC definition)
**Patients† (lesions)**	12 (19)	5	26 (A) 12 (B)	16	18 (19)	13	29 (group A)	30	154 (162)	45* (*central +SABR)
**Histology**	NSCLC (primary/mets)	NSCLC (OMD/inoperable)	Mix	Mix (OMD/primary NCLC	Mix (OMD/OPD)	Mix (primary/mets)	Mix (78% NSCLC)	Primary NSCLC	Mix	Primary NSCLC (inoperable)
**Dose regimens, Gy/#**	40/8 (30-60/5-8) (BED_10_ 48-105)	50/5 (BED_10_ 100)	60/8 (50-60/5-15) (BED_10_ 75-105)	50-60/10 (BED_10_ 75-90)	50/5 (35-60/5)	60/15	56/8 (BED_10_ 95.2)	60/8 (BED_10_ 105)	30-55 Gy/5 (BED_10_ 48-115)	60/8 (BED_10_ 105)
**Survival Parameters *(calculate from SABR)***										
**OS, Median, %**	15m 1y: 80%	1y:60%	1&2y GroupA: 89 GroupB:81 & 76	2y 63%	1y-75 %, 2y-66 (no distinction central vs UC)	NR	1y 81; 2y 58	3y 72.5	44m 1y 78%; 2y 67%	1y 95; 2y 78
**LC, %**	94.7%	1y: 80	2y:84;3y:78 groupA: 2y:91; 3y:81	2y 93	1y 94; 2y 86%	6m: 92.3%	85 (1y); 83 (2 and 3y)	89.6 at 3y	95.2 (2y 89%; 3y 86%	87.6% 3y (no distinction central vs UC)
**G3-5 toxicity, %**	0%	20 (1 patient G3)	4.3% (G3) (no distinction by groups)	19% no death-related	0%	0%	33.8 (15.4% death-related)	6.7 (3y)	1y 6.7%, 2y 8.4%; 3y 9.4%	13 (1 G5 in UC lesion)

*Abbreviations:* BED: Biological Equivalent Dose (alpha/beta of 10);ITV: Interval Target Volume; LC: Local Control; Linac: linear accelerator; LPFS: Local Progression Free Survival; MRIdian®=6 MV 0.35T MRI-Linac ViewRay Systems^;,^NR: no reported, OS: NSCLC: Non-Small Cell Lung Cancer; Overall Survival; PBT: Proximal Bronchial Tree; PFS: Progression Free Survival; PTV: Planning Target Volume; RMM: respiratory motion management; SABR: Stereotactic Ablative Radiation Therapy; UC: Ultracentral.

#fractions, y: year(s).

†Only UCs number expressed.

**LUSTRE trial no specified number neither definition nor stratified outcomes per UC lesions.

The median simulated conventional-SABR PTV was significantly larger than the SMART PTV (30.4 cm^3^ vs 10.1 cm^3^, *P* < .001), with a significant increase in the median volume of overlap with ultracentral OARs (4.7 cm^3^ vs 0.85 cm^3^, *P* < .001) (Table 2). Furthermore, when applying the simulated conventional-SABR PTV, 6 lesions overlapped with an additional ultracentral OAR compared to the SMART PTV.

## Discussion

Our study sought to highlight the promising outcomes and safety profile of SMART in the treatment of a cohort of patients all with a histologic diagnosis of NSCLC and ultracentral thoracic metastases. With a median follow-up of 28 months, SMART demonstrated both excellent LC and a low toxicity profile. This compares favorably with nonadaptive SABR series, which have reported severe toxicity rates, including treatment-related deaths, ranging from 7% to 34% for ultracentral tumors.^4^, ^5^, ^6^, ^7^^,^^18^ The absence of grade 3 to 5 acute or late toxicities and the low incidence of acute grade 1 to 2 toxicity (54% and 18%, respectively) underscores the safety of SMART in breath-hold with gated beam delivery in this high-risk cohort. Additionally, mandatory peer review of target and OAR volumes, and acceptance of PTV coverage compromise to respect OAR constraints, likely contributed to these favorable safety outcomes. Despite individualized prescription doses to meet nationally agreed OAR constraints for mediastinal structures, our analysis demonstrated LC rates comparable to conventional nonadaptive SABR studies.^4^, ^5^, ^6^, ^7^ The observed median OS of 20 months is encouraging, particularly given that 73% of patients had a history of metastatic disease prior to SMART and underscores the importance of careful patient selection for metastasis directed therapy in multidisciplinary tumor board discussions.

Previous dose–response modeling has shown a significant correlation between PTV overlap with the mainstem bronchus or trachea and grade ≥ 3 toxicity.^18^ By employing breath-hold delivery with automated beam gating, SMART obviates the need for an ITV and permits smaller PTV margins. Real-time MR imaging enables continuous monitoring during breath-hold and allows for immediate detection of intrafractional tumor drift, ensuring that beam delivery is automatically paused if the target moves outside the defined gating boundary.^19^

Our data showing reduced PTV overlap with ultracentral OARs compared to simulated conventional-SABR PTVs are consistent with prior findings.^20^ These results suggest that daily adaptive radiation therapy with breath-hold, using SMART, may broaden the therapeutic window for patients with lung tumors, enabling an isotoxic treatment approach while maintaining or improving target coverage. Both daily adaptive planning and breath-hold delivery with real-time gated beam control are integral components of SMART, each contributing to motion management and dose optimization, and likely playing complementary roles in achieving the favorable toxicity profile observed in this study.

It should be noted that our cohort consisted predominantly of patients with OMD or OPD, whereas many published nonadaptive conventional SABR series primarily included patients with early-stage primary lung cancer. The competing risk of death from systemic progression in our population may confound LC outcomes, and comparisons to early-stage cohorts should therefore be interpreted with caution. We acknowledge several limitations in our study, including its retrospective design, small cohort size, and relatively limited median follow-up. Although the median follow-up was 28 months, late toxicities related to stereotactic radiation therapy may emerge beyond 2 years, and longer observation periods may be necessary to fully capture such events. Additionally, the inclusion of a large proportion of patients referred from external centers introduces heterogeneity in ST timing, follow-up imaging, and toxicity reporting, representing a potential source of bias.

## Conclusions

Our analysis demonstrates that hypofractionated SMART with daily online adaptation for ultracentral NSCLC achieved comparable LC to conventional nonadaptive SABR, with a safer toxicity profile. These findings support the consideration of SMART as a safer and effective treatment option for this challenging subgroup of thoracic tumors.

## Disclosures

The authors declare that they have no known competing financial interests or personal relationships that could have appeared to influence the work reported in this paper.

## Appendix Supplementary materials

Lung patient 1 - cine video Image, video 1

Lung patient 6 - cine video Image, video 2

Lung patient 11 - cine video Image, video 3

Supplementary Table S1 Image, application 4

## Footnotes

Supplementary material associated with this article can be found in the online version at doi:10.1016/j.adro.2025.101906.
