# Supplementary material for: Safety and Efficacy of Stereotactic Magnetic Resonance-Guided Adaptive Radiation Therapy (SMART) for Ultracentral Metastases in Non-Small Cell Lung Cancer
Source: Adv Radiat Oncol. 2025 Sep 18;10(12):101906. doi: 10.1016/j.adro.2025.101906 (PMC12593609; doi:10.1016/j.adro.2025.101906)
Supplement: Supplementary Table S1 [file mmc4.docx]

**Supplementary appendix**

| **Table S1. Systemic therapy details** | | | | |
| --- | --- | --- | --- | --- |
| **Pts.** | **ST prior SMART** | **Interval time (months)** | | **ST post SMART** |
|  |  | **ST to SMART** | **SMART to ST restart** |  |
| **1** | Osimertinib | 0.5 | 0.25 | Osimertinib |
| **2** | None | NA | 2 | Pembrolizumab, Pemetrexed, Carboplatin |
| **3** | Osimertinib, Pemetrexed | 0.75 | 0.5 | Osimertinib |
| **4** | Brigatinib | 0.5 | 0.5 | Brigatinib |
| **5** | Brigatinib | 0.5 | 0.5 | Brigatinib |
| **6** | None | NA | NA | None |
| **7** | None | NA | 6 | Pembrolizumab, Pemetrexed, Carboplatin |
| **8** | Atezolizumab | 0.75 | 1.5 | Atezolizumab |
| **9** | Pembrolizumab | 0.75 | 0.5 | Pembrolizumab |
| **10** | Brigatinib | 0.5 | 1 | Lorlatinib |
| **11** | None | NA | NA | None |
| **12** | Carboplatin, Etoposide | 0.75 | 2 | Carboplatin, Etoposide |
| *NA: Not applicable; Pts: patients; SMART: Stereotactic MR-guided Adaptive Radiotherapy; ST: Systemic Therapy* | | | | |
